# Resveratrol Mitigates High Glucose-Induced Inflammation in Astroglial Cells

**DOI:** 10.3390/metabo15120771

**Published:** 2025-11-28

**Authors:** Vanessa Sovrani, Filipe Renato Pereira Dias, Rômulo Rodrigo de Souza Almeida, Krista Minéia Wartchow, Nícolas Manzke Glänzel, Ester Rezena, Carlos-Alberto Gonçalves, Guilhian Leipnitz, Larissa Daniele Bobermin, André Quincozes-Santos

**Affiliations:** 1 Programa de Pós-Graduação em Ciências Biológicas: Bioquímica, Instituto de Ciências Básicas da Saúde, Universidade Federal do Rio Grande do Sul, Porto Alegre 90610-264, RS, Brazil; vanessasovrani@gmail.com (V.S.); filipe.dias@ufrgs.br (F.R.P.D.); rrsa25@gmail.com (R.R.d.S.A.); kristawartchow@gmail.com (K.M.W.); nicolas.glanzel@gmail.com (N.M.G.); ester.rezena@ufrgs.br (E.R.); casg@ufrgs.br (C.-A.G.); guilhian@ufrgs.br (G.L.); andrequincozes@ufrgs.br (A.Q.-S.); 2 Programa de Pós-Graduação em Neurociências, Instituto de Ciências Básicas da Saúde, Universidade Federal do Rio Grande do Sul, Porto Alegre 90610-264, RS, Brazil; 3 Departamento de Bioquímica, Instituto de Ciências Básicas da Saúde, Universidade Federal do Rio Grande do Sul, Porto Alegre 90610-264, RS, Brazil

**Keywords:** C6 astroglial cells, high glucose levels, glioprotection, neuroinflammation, resveratrol

## Abstract

**Background/Objectives:** Changes in glucose metabolism impact central nervous system (CNS) homeostasis and, consequently, can lead to cognitive impairment and an increased risk for neurodegenerative and neuropsychiatric disorders. Astrocytes are glial cells that act as key regulators of brain glucose metabolism, thus representing important cellular targets for studies of different pathophysiological conditions, including hyperglycemia. Resveratrol, a natural polyphenol, has emerged as a potential protective strategy against diabetes and its complications; however, its glioprotective effects remain unclear. Based on these observations, we evaluated whether resveratrol could modify the inflammatory response in astroglial cells exposed to experimental hyperglycemic conditions. **Methods:** After reaching confluence, C6 astroglial cells were pre-incubated with 10 µM resveratrol in serum-free DMEM with 6 mM glucose for 24 h. The medium was then replaced with serum-free DMEM containing 12 mM glucose and 10 µM resveratrol for another 24 h. Controls were maintained in 6 mM glucose. Analyses included cell viability, metabolic activity, glucose and glutamate uptake, cytokine quantification by ELISA, and gene expression by RT-qPCR. **Results:** We show that high glucose levels modulate glucose and glutamate metabolism, and increase neuroinflammation, through the modulation of inflammatory mediators. In addition, high glucose upregulated the gene expressions of inducible nitric oxide synthase (iNOS), nuclear factor κB (NFκB), cyclooxygenase 2 (COX2), and Toll-like receptor 4 (TLR4) while decreasing mRNA levels of NLR family pyrin domain containing 3 (NLRP3) and peroxisome proliferator-activated receptor gamma coactivator 1-alpha (PGC-1α). However, resveratrol was able to prevent most of these effects, particularly the high glucose-triggered inflammatory response. Resveratrol also modulated heme oxygenase 1 (HO-1) and nuclear factor erythroid-derived 2-like 2 (Nrf2), important targets associated with cellular protection. **Conclusions:** Our findings reinforce resveratrol as a potential glioprotective strategy against diabetes-related brain toxicity.

## 1. Introduction

Glucose is a critical energy substrate for brain function; as such, glucose metabolism disorders, including hyperglycemia, strongly impair central nervous system (CNS) homeostasis. In astrocytes, alterations in glucose levels can result in neurotoxicity and/or gliotoxicity [1,2] and, consequently, can lead to cognitive impairment and an increased risk for neurodegenerative and neuropsychiatric disorders [3]. Accordingly, neuroinflammation has been shown to play a significant role in the pathophysiology of glucose metabolism disorders [1,4], since high glucose levels can induce the release of pro-inflammatory cytokines in astrocytes [5,6], which in turn, can induce glial reactivity, neuronal damage, and blood–brain barrier dysfunction [1]. In addition, hyperglycemia enhances brain susceptibility to neuroinflammation via astrocyte reprogramming [7].

Based on these findings, astrocytes have become cellular targets for studies of pathophysiological processes and preventive strategies for glucose metabolism disorders in the CNS [8,9]. Among the glioprotective strategies explored for the prevention and treatment of several pathologies, natural molecules have emerged as promising candidates across a number of areas [1]. Resveratrol (RSV) is a polyphenol of the stilbene family that is synthesized by a variety of plants, such as grapes, peanuts, and berries [10,11]. RSV is a well-known phenolic and antioxidant compound that prevents oxidative damage in various pathological conditions. In diabetic animal models, for example, RSV has demonstrated protective effects against cognitive decline and degeneration [12,13], including attenuation of astrocytic activation in the hippocampus [14]. In vitro, RSV treatment induces glial responses, including by reducing microglial activation, decreasing inflammatory mediators and maintaining the redox status after different insults in astroglial cells [15,16]. With regard to the protective role of RSV, long-term administration in vivo has been shown to improve cognitive performance in the elderly male rat model, possibly mediated by the anti-inflammatory and antioxidant effects of RSV [17].

The effects of RSV on astroglial cells, in high-glucose conditions, however, have not been widely explored, warranting further investigation for better understanding of the mechanisms by which RSV may attenuate the neurological complications associated with glucose metabolism disorders. As such, the aim of this study was to investigate whether RSV can mitigate high glucose-induced inflammation. For this, we evaluated the effects of 48 h RSV exposure on C6 astroglial cells, an astrocyte-like cell line, when cultured under high-glucose conditions, focusing on the inflammatory response and the underlying signaling mechanisms associated with its glioprotective activity.

## 2. Methods

### 2.1. C6 Astroglial Cell Culture and Treatments

C6 astroglial cells were obtained from the American Type Culture Collection (Rockville, MA, USA) and were maintained, essentially, according to our previous publication [18]. These cells have been widely used to study astroglial functions (oxidative/nitrosative and inflammatory responses, glutamate uptake, glutamine synthetase activity, among others) and associated signaling pathways comparable to those observed in primary astrocyte cultures, even under conditions of metabolic stress [5,19]. The cells were seeded in flasks and cultured in Dulbecco’s modified Eagle’s medium containing 6 mM glucose (DMEM; Gibco, Waltham, MA, USA), pH 7.4, supplemented with 5% fetal bovine serum (FBS; Gibco, Waltham, MA, USA), 2.5 mg/mL amphotericin B (Gibco, Waltham, MA, USA) and 0.05 mg/mL gentamicin (Gibco, Waltham, MA, USA), at 37 °C in an atmosphere of 5% CO_2_. Exponentially growing cells were detached from the culture flasks using 0.05% trypsin/EDTA (Gibco, Waltham, MA, USA) and seeded (5 × 10^3^ cells/cm^2^) in 24- or 6-well plates. After the cells reached confluence, the culture medium was exchanged for serum-free DMEM supplemented with 6 mM glucose, and C6 astroglial cells were pre-incubated with 10 µM RSV (Sigma-Aldrich, Saint Louis, MO, USA) for 24 h at 37 °C in an atmosphere of 5% CO_2_. Afterwards, the culture medium was exchanged again for serum-free DMEM containing 12 mM glucose (high glucose). At this time, 10 µM RSV was added again and C6 astroglial cells were incubated for an additional 24 h at 37 °C in an atmosphere of 5% CO_2_. Control conditions were performed in the presence of serum-free DMEM containing 6 mM glucose.

### 2.2. MTT Reduction Assay

The MTT reduction assay was used to verify cellular viability. Cells were incubated with MTT (methylthiazolyldiphenyl-tetrazolium bromide; Sigma-Aldrich, Saint Louis, MO, USA) at a final concentration of 50 μg/mL for 30 min at 37 °C in an atmosphere of 5% CO_2_. The medium was then removed for dissolving MTT crystals in DMSO, and absorbance was measured at 560 and 650 nm [5]. The results are expressed as percentages relative to the control values.

### 2.3. Extracellular Lactate Levels

Lactate levels in the extracellular medium were quantified using a commercial kit from Bioclin (Belo Horizonte, MG, Brazil). The results are expressed in mmol/L.

### 2.4. Glucose Uptake Assay

Glucose uptake was performed as previously described [5] with some modifications. The cells were incubated at 37 °C in Hank’s balanced salt solution (HBSS). The assay started with the addition of 0.1 µCi/well D-[2,3-^3^H]-deoxy-glucose for 15 min. The incubation was stopped by removing the medium and rinsing the cells twice with ice-cold HBSS. The cells were then lysed in a 0.5 M NaOH solution. Glucose uptake was calculated by subtracting the nonspecific uptake, obtained using the glucose transporter inhibitor, cytochalasin B (25 µM), from the total uptake. Radioactivity was measured using a scintillation counter. The results are expressed as nmol/mg protein/min.

### 2.5. Glutamate Uptake Assay

Glutamate uptake was measured as previously described [18]. Briefly, C6 cells were incubated at 37 °C in a Hank’s balanced salt solution (HBSS) containing (in mM): 137 NaCl, 5.36 KCl, 1.26 CaCl_2_, 0.41 MgSO_4_, 0.49 MgCl_2_, 0.63 Na_2_HPO_4._7H_2_O, 0.44 KH_2_PO_4_, 4.17 NaHCO_3_ and 5.6 glucose, adjusted to pH 7.4. The assay was started by the addition of 0.1 mM L-glutamate and 0.33 μCi/mL L-[2,3-^3^H] glutamate. The incubation was stopped after 10 min by removal of the medium and rinsing the cells twice with ice-cold HBSS. The cells were then lysed in a solution containing 0.5 M NaOH. Radioactivity was measured in a scintillation counter. Sodium-independent uptake was determined using N-methyl-D-glucamine instead of NaCl. Sodium-dependent glutamate uptake was obtained by subtracting the nonspecific uptake from the total to obtain the specific uptake. The results are expressed as nmol/mg protein/min.

### 2.6. RNA Extraction and Quantitative RT-PCR

Total RNA was isolated from astroglial C6 cultures using TRI Reagent (Sigma-Aldrich, Saint Louis, MO, USA). cDNA was synthesized from extracted RNA (1 μg) using the High-Capacity cDNA Reverse Transcription Kit (Applied Biosystems/Thermo Fisher Scientific, Waltham, MA, USA). Quantitative PCR determination of the messenger RNAs (mRNAs) encoding cyclooxygenase 2 (COX2; #Rn01483828_m1), excitatory amino acid transporter (EAAC1; #Rn00564705_m1), glucose transporter 1 (GLUT1; #Rn01417099_m1), glutamine synthetase (GS; #Rn01483107_m1), heme oxygenase 1 (HO-1; #Rn01536933_ m1), inducible nitric oxide synthase (iNOS; #Rn00561646_m1), interleukin-1β (IL-1β; #Rn00580432_m1), interleukin 1 receptor type 1 (IL1R1; #Rn00565482_m1), interleukin-10 (IL-10; #Rn00563409_m1), nuclear factor erythroid-derived 2-like 2 (Nrf2; #Rn00582415_m1), nuclear factor κB p65 subunit (NFκB p65; #Rn01502266_m1), NLR family pyrin domain containing 3 (NLRP3; #Rn04244620_m1), peroxisome proliferator-activated receptor gamma coactivator 1-alpha (PGC-1α; #Rn00580241_m1), Toll-like receptor 2 (TLR2; #Rn02133647_s1), Toll-like receptor 4 (TLR4; #Rn00569848_m1), tumor necrosis factor-α (TNF-α; #Rn99999017_m1), and tumor necrosis factor receptor 1 (TNFR1; #Rn01492348_m1) was performed using the TaqMan real-time RT-PCR system (QuantiStudio 1, Applied Biosystems, Waltham, MA, USA) with inventory primers and probes purchased from Applied Biosystems (Thermo Fisher Scientific, Waltham, MA, USA), as referred for each gene. Target mRNA levels were normalized to β-actin (#Rn00667869_m1) levels. The results were analyzed employing the 2^−ΔΔCt^ method [20].

### 2.7. Measurement of Cytokine Levels

The extracellular levels of the cytokines, tumor necrosis factor α (TNF-α), interleukin-1β (IL-1β), and interleukin-10 (IL-10), were measured using rat ELISA kits according to the manufacturer’s instructions. The assay ranges for the kits are: 16 to 2000 pg/mL for TNF-α (Invitrogen, Waltham, MA, USA. Catalog #88-7340-22); 31.3 to 2000 pg/mL for IL-1β (Invitrogen, Waltham, MA, USA. Catalog #BMS630); 15.6–1000 pg/mL for IL-10 (Invitrogen, Waltham, MA, USA. Catalog #BMS629). The results are expressed in pg/mL.

### 2.8. Protein Content

The protein content was determined by the method of Lowry et al. [21] using bovine serum albumin (BSA) as a standard.

### 2.9. Statistical Analysis

Data are presented as means ± S.D. of at least six independent experiments. The normal distribution was confirmed by the Shapiro–Wilk test, and differences among groups were analyzed statistically using a one-way analysis of variance (ANOVA), followed by Tukey’s test. *p* values < 0.05 were considered significant. All analyses were performed using Graphpad Prism software version 10 (GraphPad 390 Software, Inc., La Jolla, CA, USA). *a* indicates difference from control group; *b* indicates difference from high glucose.

## 3. Results

### 3.1. Resveratrol Attenuates High-Glucose–Induced Alterations in Glucose but Not Glutamate Metabolism in C6 Astroglial Cells

**Figure 1:**
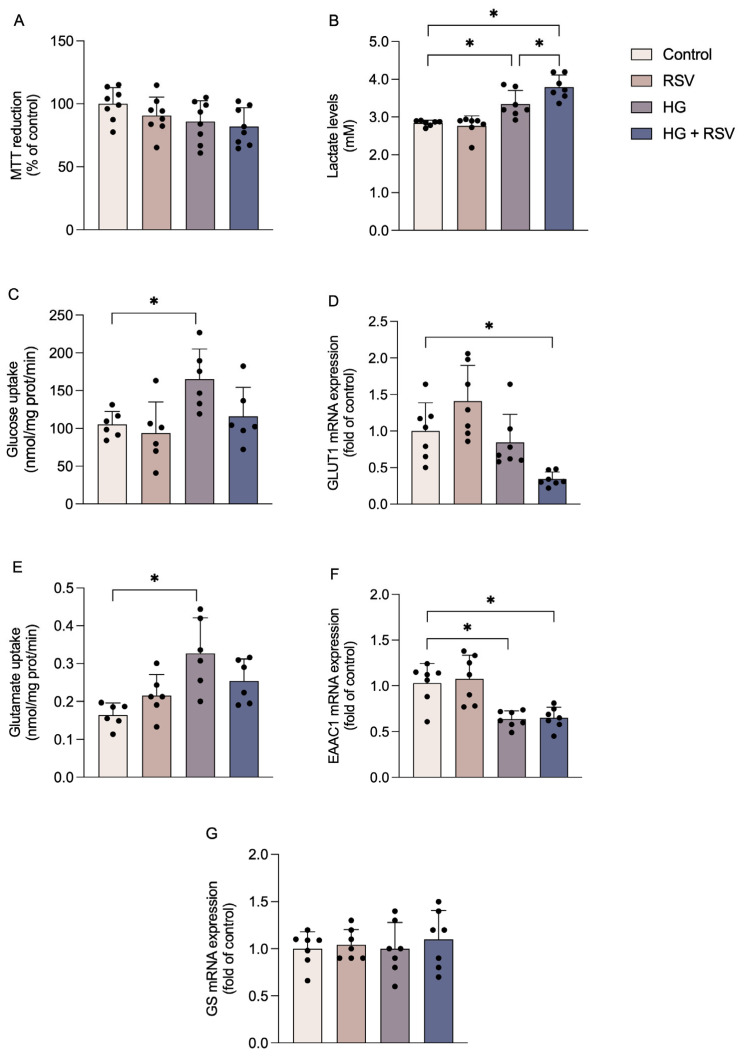
Effects of high glucose and resveratrol on viability and metabolic functions in C6 astroglial cells. Cells were pre-treated with resveratrol (RSV; 10 µM) for 24 h and subsequently exposed to high glucose medium (HG; 12 mM) in the presence or absence of RSV (10 µM) for an additional 24 h. MTT reduction (**A**), extracellular lactate levels (**B**), glucose uptake (**C**), glutamate uptake (**E**), the mRNA expressions of the genes encoding GLUT1 (**D**), EAAC1 (**F**), and GS (**G**), were evaluated. The results represent the means ± S.D., analyzed by one-way ANOVA. Values of *p* < 0.05 were considered significant (6–8 independent cultures with at least duplicate treatments, as shown by the dispersion of the data points in the graphs). Control: DMEM, 6 mM glucose; RSV: DMEM, 6 mM glucose + 10 µM RSV; HG: DMEM, 12 mM glucose; HG + RSV: DMEM, 12 mM glucose + 10 µM RSV. * indicates a statistical difference between the groups (*p*-values are provided in the Results).

Initially, we assessed the effects of RSV and high glucose on cell viability. A 24 h exposure to high glucose medium was selected to reproduce an acute hyperglycemic condition capable of inducing oxidative and inflammatory responses in neural cells [6,22,23]. Resveratrol was applied as a pre-treatment and maintained during the high-glucose exposure to allow for the activation and persistence of its protective effects throughout the metabolic challenge. Neither of RSV nor high glucose treatments affected MTT reduction in C6 astroglial cells (Figure 1A; F_(3,28)_ = 2.178, *p* = 0.1128). Subsequently, we evaluated some parameters associated with glucose metabolism, including extracellular lactate levels, which were increased at high glucose (F_(3,24)_ = 21.22, adjusted *p* value = 0.0116) and with high glucose plus RSV (F_(3,24)_ = 21.22, adjusted *p* value < 0.0001) (Figure 1B). In addition, high glucose levels increased glucose uptake (Figure 1C; F_(3,20)_ = 4.650, adjusted *p* value = 0.0405), while in the presence of RSV, glucose uptake was maintained near to that of control conditions (F_(3,20)_ = 4.650, adjusted *p* value = 0.9548, when comparing the control group with the high glucose plus RSV group). The expression of GLUT1 was not affected by high glucose (F_(3,24)_ = 9.990, adjusted *p* value = 0.8614) or by RSV (F_(3,24)_ = 9.990, adjusted *p* value = 0.1880) alone, but was decreased in the presence of high glucose plus RSV, compared to control conditions (Figure 1D; F_(3,24)_ = 9.990, adjusted *p* value = 0.014).

Regarding glutamate metabolism, glutamate uptake was increased in the high-glucose group (Figure 1E; F_(3,20)_ = 6.943, adjusted *p* value = 0.0014). In contrast, EAAC1 (Figure 1F), the main glutamate transporter expressed by C6 astroglial cells, was downregulated in the groups exposed to high glucose (F_(3,24)_ = 11.59, adjusted *p* value = 0.0029), even in the presence of RSV (F_(3,24)_ = 11.59, adjusted *p* value = 0.0040). The expression of GS, which converts glutamate to glutamine in glial cells, did not demonstrate significant change (Figure 1G; F_(3,24)_ = 0.2745, *p* value = 0.8432).

In summary, high glucose seems to alter both glucose and glutamate metabolism in C6 astroglial cells, and resveratrol partially prevented the glucose-related metabolic alterations but did not prevent the glutamate metabolism changes.

### 3.2. Resveratrol Prevents the High Glucose-Triggered Inflammatory Response in Astroglial Cells

**Figure 2:**
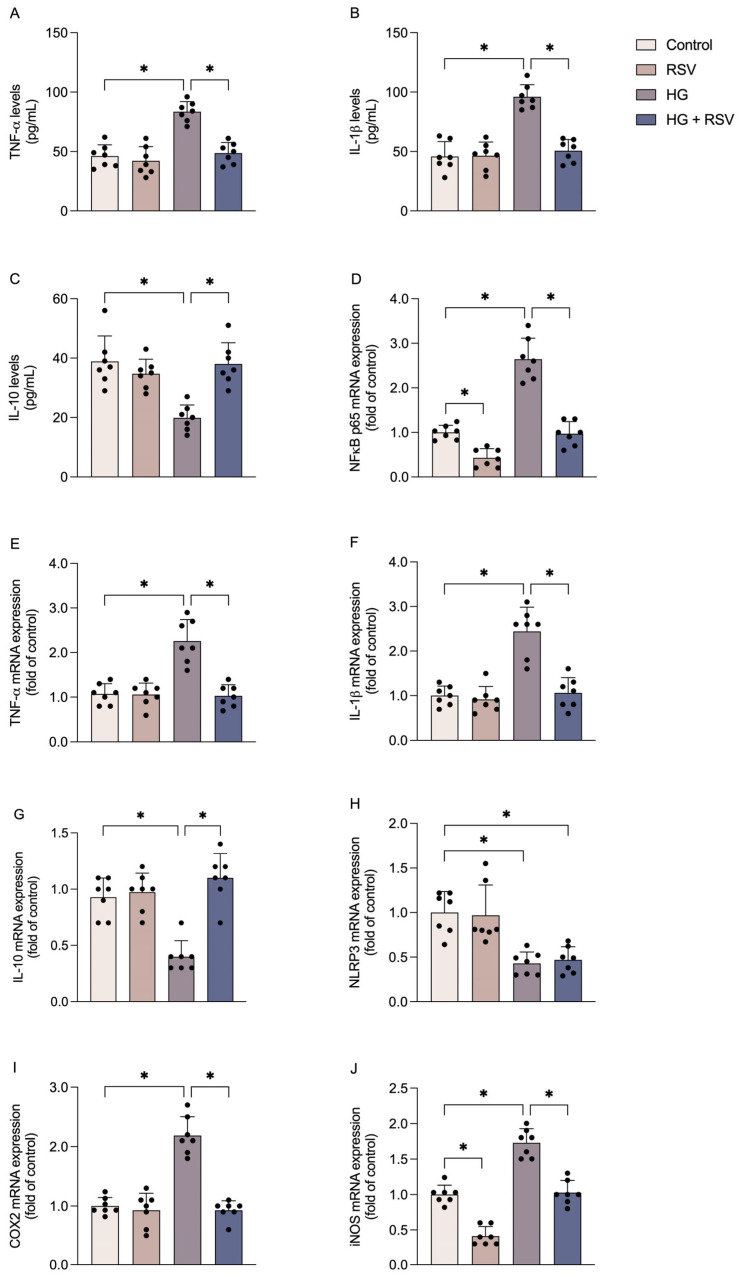
Effects of high glucose and resveratrol on inflammatory signaling in C6 astroglial cells. Cells were pre-treated with resveratrol (RSV; 10 µM) for 24 h and subsequently exposed to high glucose medium (HG; 12 mM) in the presence or absence of RSV (10 µM) for an additional 24 h. The levels of TNF-α (**A**), IL-1β (**B**), and IL-10 (**C**), and the mRNA expression of the genes encoding NFκB p65 (**D**), TNF-α (**E**), IL-1β (**F**), IL-10 (**G**), NLRP3 (**H**), COX2 (**I**), and iNOS (**J**) were evaluated. The results represent the means ± S.D., analyzed by one-way ANOVA. Values of *p* < 0.05 were considered significant (n = 7 independent cultures with at least duplicate treatments, as shown by the dispersion of the data points in the graphs). Control: DMEM, 6 mM glucose; RSV: DMEM, 6 mM glucose + 10 µM RSV; HG: DMEM, 12 mM glucose; HG + RSV: DMEM, 12 mM glucose + 10 µM RSV. * indicates a statistical difference between the groups (*p*-values are provided in the Results).

High glucose conditions and RSV modulated the inflammatory response in astroglial cells. Specifically, high glucose increased extracellular levels of the pro-inflammatory cytokines TNF-α (Figure 2A; F_(3,24)_ = 26.55, adjusted *p* value < 0.0001) and IL-1β (Figure 2B; F_(3,24)_ = 34.58, adjusted *p* value < 0.0001), while decreasing the levels of the anti-inflammatory cytokine, IL-10 (Figure 2C; F_(3,24)_ = 12.98, adjusted *p* value < 0.0001). RSV, in turn, prevented all of these effects of high glucose (adjusted *p* value ≤ 0.0001 for all cytokines evaluated, when comparing the HG and HG + RSV groups).

NF-κB p65 plays a crucial role in regulating neuroinflammation, and its transcriptional levels were markedly upregulated under high glucose conditions (Figure 2D; F_(3,24)_ = 70.91, adjusted *p* value < 0.0001). RSV also prevented this effect (adjusted *p* value < 0.0001), in addition to decreasing the mRNA levels of NFκB p65 under control conditions (adjusted *p* value = 0.0081). We then evaluated the expression of transcriptional targets of NFκB, including the inflammatory cytokines. The changes in cytokine gene expression that were induced by high glucose were consistent with the alterations observed in their extracellular levels, with upregulation of the genes encoding TNF-α (Figure 2E; F_(3,24)_ = 24.38, adjusted *p* value < 0.0001) and IL-1β (Figure 2F; F_(3,24)_ = 27.33, adjusted *p* value < 0.0001), and downregulation of the gene encoding IL-10 (Figure 2G; F_(3,24)_ = 21.39, adjusted *p* value < 0.0001). These changes were also prevented by RSV treatment (adjusted *p* value < 0.0001).

In addition to cytokines, other transcriptional targets of NFκB were evaluated. The NLRP3 inflammasome processes IL-1β for release, thus playing a central role in neuroinflammation. However, NLRP3 expression was decreased (Figure 2H; F_(3,24)_ = 12.90, adjusted *p* value = 0.0005) under high-glucose conditions, and this reduction was not prevented by RSV (adjusted *p* value = 0.9876, when comparing the HG and HG + RSV groups). In contrast, the gene expression of COX2 (Figure 2I) and iNOS (Figure 2J), other classical inflammatory markers, followed a similar profile to that observed for the cytokines, being upregulated by high glucose (F_(3,24)_ = 46.29, adjusted *p* value < 0.0001 for COX2 and F_(3,24)_ = 78.38, adjusted *p* value < 0.0001 for iNOS) and maintained at levels similar to those of the control by RSV treatment (adjusted *p* value < 0.0001 for both genes). Additionally, RSV downregulated iNOS expression under basal conditions (adjusted *p* value < 0.0001).

**Figure 3:**
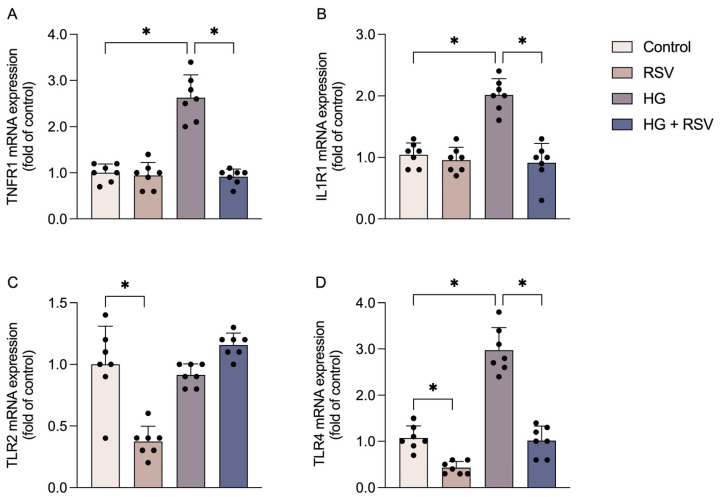
High glucose and resveratrol modulate the gene expression of membrane receptors that trigger intracellular inflammatory pathways. Cells were pre-treated with resveratrol (RSV; 10 µM) for 24 h and subsequently exposed to high-glucose medium (HG; 12 mM) in the presence or absence of RSV (10 µM) for an additional 24 h. The mRNA expressions of genes encoding TNFR1 (**A**), IL1R1 (**B**), TLR2 (**C**), and TLR4 (**D**) were evaluated. The results represent the means ± S.D., analyzed by one-way ANOVA. Values of *p* < 0.05 were considered significant (n = 7 independent cultures with at least duplicate treatments, as shown by the dispersion of the data points in the graphs). Control: DMEM, 6 mM glucose; RSV: DMEM, 6 mM glucose + RSV 10 µM; HG: DMEM, 12 mM glucose; HG + RSV: DMEM, 12 mM glucose + RSV 10 µM. * indicates a statistical difference between the groups (*p*-values are provided in the Results).

The activation of these inflammatory pathways may be modulated by receptors located on the cell membrane, including cytokine receptors and pattern-recognition receptors, which detect external inflammatory stimuli. The expressions of TNFR1 (Figure 3A; F_(3,24)_ = 50.96, adjusted *p* value < 0.0001) and IL1R1 (Figure 3B; F_(3,24)_ = 31.37, adjusted *p* value < 0.0001) were increased by high glucose, and this effect was prevented by RSV (adjusted *p* value < 0.0001 for both genes). With regard to TLR2 (Figure 3C) and TLR4 (Figure 3D), RSV alone reduced the expression of both receptors (F_(3,24)_ = 25.10, adjusted *p* value < 0.0001 for TLR2 and F_(3,24)_ = 79.23, adjusted *p* value = 0.0063 for TLR4). High-glucose conditions increased only the expression of TLR4 (adjusted *p* value < 0.0001), and this effect was prevented by RSV treatment (adjusted *p* value < 0.0001).

Taken together, these results indicate that high glucose triggers a pro-inflammatory response in C6 astroglial cells, characterized by NFκB activation and altered expression of cytokines, inflammasome, and receptor-related genes. Resveratrol prevented most of these changes, maintaining cytokine balance and suppressing inflammatory signaling.

### 3.3. Signaling Pathways Associated with the Anti-Inflammatory Effects of Resveratrol

**Figure 4:**
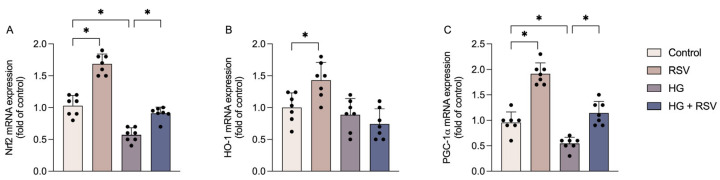
Resveratrol attenuated high glucose-induced downregulation of cytoprotective anti-inflammatory pathways. Cells were pre-treated with resveratrol (RSV; 10 µM) for 24 h and subsequently exposed to high glucose medium (HG; 12 mM) in the presence or absence of RSV (10 µM) for an additional 24 h. The mRNA expressions of genes encoding Nrf2 (**A**), HO-1 (**B**), and PGC-1α (**C**) were evaluated. The results represent the means ± S.D., analyzed by one-way ANOVA. Values of *p* < 0.05 were considered significant (n = 7 independent cultures with at least duplicate treatments, as shown by the dispersion of the data points in the graphs). Control: DMEM, 6 mM glucose; RSV: DMEM, 6 mM glucose + 10 µM RSV; HG: DMEM, 12 mM glucose; HG + RSV: DMEM, 12 mM glucose + 10 µM RSV. * indicates a statistical difference between the groups (*p*-values are provided in the Results).

Finally, we assessed classical pathways associated with glioprotection. The expression of the Nrf2 transcription factor (Figure 4A) was significantly reduced following high-glucose exposure (F_(3,24)_ = 83.95, adjusted *p* < 0.0001). RSV prevented this reduction (adjusted *p* = 0.0005) and, in addition, significantly upregulated Nrf2 under basal conditions (adjusted *p* < 0.0001). Given that HO-1 is a well-known downstream target of Nrf2, we next evaluated the expression of this enzyme. Although HO-1 expression (Figure 4B) remained unchanged under high-glucose conditions (F_(3,24)_ = 9.687, adjusted *p* value = 0.8243), it was upregulated by RSV alone (adjusted *p* value = 0.0196).

Accordingly, we also observed changes in the expression of PGC-1α (Figure 4C), a transcription co-activator that is functionally linked to Nrf2 signaling. The mRNA expression of PGC-1α was decreased by high glucose (F_(3,24)_ = 58.95, adjusted *p* value = 0.0034). In the presence of RSV, high glucose no longer decreased the expression of PGC-1α (adjusted *p* value < 0.0001), and its expression was increased under basal conditions (adjusted *p* value < 0.0001).

## 4. Discussion

Hyperglycemia impacts brain energy homeostasis, leading to alterations in neurotransmission, synaptic plasticity, and cognitive functions [1,24]. Astrocytes play critical metabolic roles in the CNS, including in glucose uptake and the transfer of metabolic substrates, processes that are maintained by tight regulatory mechanisms [25,26,27]. In this context, the metabolic activity of astroglial cells, including the C6 astrocyte-like cell line used in this study, may be reprogrammed in response to changes in glucose availability [5,28,29]. This is consistent with our experimental model, where high glucose induced metabolic and functional alterations, including inflammatory activation and downregulation of anti-inflammatory genes. These observations support the notion that astroglial cells are key cellular players in the CNS response to hyperglycemic stress, and may serve as relevant targets for protective strategies. RSV, by modulating inflammatory and metabolic pathways, emerges as a potential multi-target compound for mitigating the detrimental effects of hyperglycemia on astroglial function. Notably, RSV has been reported to cross the blood–brain barrier (BBB) in rodents after oral administration [30,31]. Given the low brain bioavailability of resveratrol due to rapid metabolism, nanotechnology-based strategies are increasingly being explored to enhance brain delivery by improving stability and facilitating blood–brain barrier penetration [32,33]. Consequently, investigating the biological effects of resveratrol in glial cells is highly relevant for developing neuroprotective strategies.

In our study, high glucose stimulated glucose uptake in astroglial cells without increasing GLUT1 expression, suggesting enhanced transporter activity due to substrate availability. Although GLUT1 is considered to be the primary route for glucose uptake [34], GLUT1 knock-out mice maintain normal resting astrocytic glucose levels [35]. RSV reduced GLUT1 mRNA under high glucose and maintained glucose uptake at control levels, indicating its ability to prevent excessive glucose entry during hyperglycemia. These findings align with previous reports showing that RSV decreases GLUT1-mediated glucose uptake [36].

Astroglial cells are primarily glycolytic and produce lactate as a key metabolic product [27]. Consistent with this metabolic profile and the observed increase in glucose uptake, we detected elevated extracellular lactate levels in cells exposed to high-glucose conditions. Interestingly, RSV did not prevent this increase in lactate, suggesting that RSV may act mainly by limiting glucose transport rather than affecting metabolism in C6 cells. Notably, lactate has been increasingly considered as a signaling factor that may even regulate glucose uptake; elevated extracellular lactate levels may signal the need for enhanced glucose uptake to sustain oxidative metabolism and support brain energy demands [37]. Therefore, the increase in lactate levels may indicate a protective role under stress conditions, as suggested in brain disorders such as diabetes and ischemia-reperfusion injury [37,38], and RSV may contribute to this effect.

In addition to glucose metabolism, astrocytes participate in the glutamate-glutamine cycle, an important metabolic cooperation between astrocytes and neurons [39]. Previous studies have shown increased glutamate uptake in astroglial cells exposed to high glucose [5], which is consistent with our data. However, we observed a reduction in EAAC1 mRNA levels following high-glucose exposure, regardless of RSV treatment. Although changes in glutamate transporter activity may be associated with glycation [40] or changes in membrane translocation [41], the downregulation of EAAC1 could represent a compensatory mechanism in response to altered transporter function. These findings suggest adjustments in glutamate uptake and metabolism under high-glucose conditions. In this context, the absence of changes in the gene expression of GS, the enzyme that converts glutamate into glutamine, raises the possibility that glutamate may be metabolized via alternative oxidative pathways [42].

Interestingly, changes in glucose and glutamate metabolism may support neuroinflammation by modulating immunometabolism in astrocytes. Immunometabolism refers to the interplay between cellular metabolism and immune function, where shifts in glucose utilization modulate inflammatory responses by affecting energy supply and biosynthetic processes [43]. Accordingly, previous studies have demonstrated that hyperglycemia and/or high glucose levels amplify neuroinflammatory responses to pathological stimuli, such as lipopolysaccharide and Zika virus [28]. In this study, we confirmed the high glucose-induced pro-inflammatory response in astroglial cells. Moreover, we demonstrate that RSV exerts a protective effect under these conditions by decreasing gene expression and/or extracellular levels of key inflammatory mediators, thus potentially attenuating hyperglycemia-induced neuroinflammation.

Given that NFκB transcriptional activity may modulate inflammation, glucose and glutamate metabolism, it may represent an integrative molecular mechanism underlying astrocyte damage [5,44]. This transcription factor regulates the inflammatory response through the activation of inflammatory genes, including cytokines, NLRP3, COX2, and iNOS [45]. More specifically, the NLRP3 inflammasome plays a key role in astrocyte responses by sensing metabolic and cellular stress, leading to IL-1β and IL-18 processing and release [46]. Under hyperglycemic conditions, NLRP3 expression and activation are typically enhanced, contributing to neuroinflammation and CNS dysfunction [47]. However, our data showed that high glucose reduced mRNA levels of NLRP3 in astroglial cells. This suggests that NLRP3 modulation may occur in a cell type-specific manner, potentially being upregulated in microglia, but not in astroglial cells [48]. Moreover, RSV has been shown to modulate NLRP3 activation [16], and it did not modify the effect of high glucose on NLRP3 expression in astroglial cells. Furthermore, while our data revealed a negative regulation of NLRP3 expression in astroglial cells under high-glucose conditions, COX2 and iNOS exhibited a gene expression pattern that was consistent with the upregulation observed for the pro-inflammatory cytokines, TNF-α and IL-1β. COX2 and iNOS are important mediators of the astrocytic inflammatory response, contributing to neuroinflammation through prostaglandin and nitric oxide production, respectively [45]. RSV also effectively prevented this response, likely through inhibition of NFκB signaling, as reported in other models of metabolic and neuroinflammatory stress [45].

The activation of NFκB, and its subsequent pro-inflammatory cascade, can be modulated by membrane receptors, including cytokine receptors and TLRs, which are able to integrate extracellular signals with intracellular inflammatory pathways [49]. The gene expression of TNFR1, IL1R1, and TLR4 was increased under high-glucose conditions, potentially altering the susceptibility of astroglial cells to inflammatory stimuli. Hyperglycemia in diabetes leads to the increased expression and activation of these inflammatory receptors in different cell types [50,51,52], but its role in astrocyte function remains underexplored. Our data show the ability of RSV to modulate both cytokine receptors and TLRs, which is critical to attenuate high glucose-driven neuroinflammation and its complications [53].

The dynamic interaction between NFκB and Nrf2 signaling pathways plays a pivotal role in coordinating metabolic and inflammatory responses, highlighting their integrated regulation during cellular adaptation to stress. While NFκB primarily regulates pro-inflammatory gene expression, Nrf2 predominantly modulates antioxidant and anti-inflammatory responses, as well as metabolic homeostasis [54]. HO-1, a key transcriptional target of Nrf2, contributes to the anti-inflammatory effects of this pathway through its enzymatic products, which attenuate NFκB activation and iNOS activity [55]. Emerging evidence suggests that Nrf2 also influences mitochondrial function by modulating the expression or activity of PGC-1α, which regulates mitochondrial biogenesis [56]. Previous studies have reported impairments in Nrf2, HO-1, and PGC-1α signaling in the brains of animals subjected to diabetes mellitus models [57,58]. RSV, in turn, has been identified as a potent inducer of Nrf2 [59]. In our study, RSV increased the gene expression of these key mediators under basal conditions and protected the downregulation of Nrf2 and PGC-1α under high-glucose stress.

The present study presents some limitations that should be acknowledged. Although the use of the C6 cell line presents inherent limitations and cannot fully reproduce the complexity of astrocytes, it constitutes an established cellular model for exploring cell signaling, gene expression, and even intrinsic astrocyte functions [60,61,62,63], often yielding results comparable to those obtained in primary astrocyte cultures, including under metabolic stresses [5,64,65]. Nevertheless, future studies employing primary astrocyte cultures (including 3D models) and in vivo models, as well as assessing time and dose-dependent effects, are necessary to confirm the present findings and further characterize the glioprotective actions of resveratrol.

**Figure 5:**
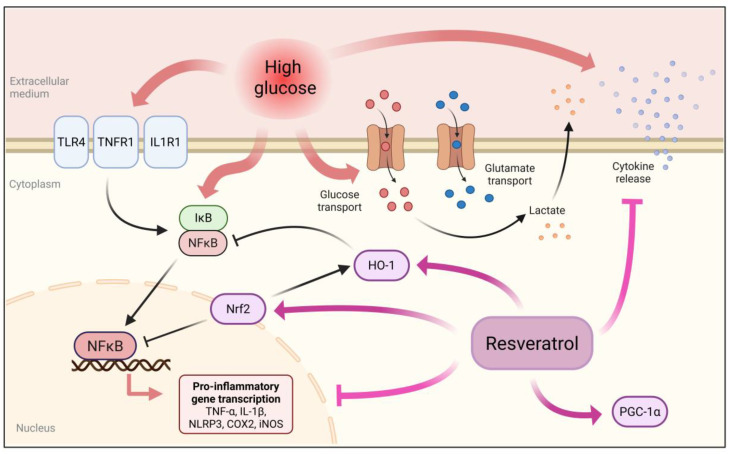
Schematic representation of high glucose-induced effects and resveratrol-mediated glioprotection in astroglial cells. High glucose levels induced a pro-inflammatory response, characterized by increased cytokine release, likely driven by activation of the NFκB pathway. Furthermore, upregulated expression of neuroinflammation-related genes, including those encoding TNF-α, IL-1β, NLRP3, COX2, and iNOS, was observed. High glucose also increased the expression of genes encoding membrane receptors such as TLR4, TNFR1, and IL1R1, which can further activate NFκB, amplifying the inflammatory process. Metabolic changes were also observed, including enhanced uptake of glucose and glutamate, along with increased lactate production. When cells were incubated in high glucose medium in the presence of resveratrol, the inflammatory response was notably prevented, possibly through activation of Nrf2 and PGC-1α. Under basal conditions, resveratrol also increased HO-1 expression that, along with Nrf2, may counteract NFκB-driven inflammation. Created in BioRender. Bobermin, L. (2025) https://BioRender.com/hwdl4gj (accessed on 8 September 2025).

In summary, we demonstrated that high glucose induces multiple changes in astroglial functions, particularly affecting metabolic and inflammatory processes (Figure 5). Considering their roles, astroglial cells have emerged as potential cellular targets for protective strategies, including against glucose disorder metabolism. Furthermore, our data showed that RSV may represent a relevant glioprotective strategy, modulating important targets for glioprotection, such as NFκB, Nrf2, and PGC-1α. Finally, bioactive compounds from plant-based foods, such as RSV, are being increasingly explored for their protective effects against metabolic complications, including diabetes-induced neuroinflammation. Cumulative preclinical evidence strongly supports that combining the established antidiabetic agent metformin with RSV can further mitigate diabetes-associated complications [66]. Therefore, RSV may serve as a promising adjuvant to conventional treatments, enhancing therapeutic outcomes in diabetes-related pathologies.

## Data Availability

The datasets used and/or analyzed during the current study are available from the corresponding author on reasonable request.
